# Clinicopathological Characteristics, Treatment Patterns, and Outcomes in Patients with Laryngeal Cancer

**DOI:** 10.3390/curroncol30040327

**Published:** 2023-04-20

**Authors:** Dejan Đokanović, Radoslav Gajanin, Zdenka Gojković, Goran Marošević, Igor Sladojević, Vesna Gajanin, Olja Jović-Đokanović, Ljiljana Amidžić

**Affiliations:** 1 University Clinical Center of the Republic of Srpska, Oncology Cllinic, 78000 Banja Luka, Republic of Srpska, Bosnia and Herzegovina; 2 University of Banja Luka, Faculty of medicine 78000 Banja Luka, Republic of Srpska, Bosnia and Herzegovina; 3 Affidea-IMC Center for Radiotherapy Banja Luka, 78000 Banja Luka, Republic of Srpska, Bosnia and Herzegovina; 4 University Clinical Center of the Republic of Srpska, Infectology Cllinic, 78000 Banja Luka, Republic of Srpska, Bosnia and Herzegovina

**Keywords:** laryngeal cancer, survival, recurrence, prognosis

## Abstract

Background: Various factors can affect the survival of patients with laryngeal cancer (LC). In this retrospective study, we assessed clinicopathological features, their prognostic value, and treatment modalities for patients with confirmed squamous cell LC. Methods: We collected patient data on demographics, clinicopathological characteristics, treatment patterns, and outcomes. The primary endpoints were overall survival (OS), disease-specific survival (DSS), disease-free survival (DFS), and locoregional control (LRC). We assessed survival using the Kaplan–Meier method and Cox regression model analyses of potential prognostic parameters. Results: After a median follow-up of 76 months, 28 (33.3%) patients had a recurrence. The median OS was 78 months, with an event recorded in 50% of patients. The DSS median was not reached (NR) with a survival rate of 72.6%, the DFS survival rate was 66.7% with median NR, and the LRC survival rate was 72.6% with median NR. After conducting a multivariate analysis of significant variables, we found that only recurrence and lymphatic invasion had an independent effect on OS and recurrence in DSS, while subsite impacted DFS and LRC. Conclusions: Survival trends were consistent with other studies, except for OS. Recurrence, lymphatic invasion, and subsite location were significant factors that impacted patient survival.

## 1. Introduction

Head and neck squamous cell carcinoma (HNSCC) is a prevalent cancer worldwide, ranking sixth among all adult cancers and accounting for 5.3% of all cancers [1]. LC is the second most common type of head and neck cancer, with 211,000 new cases and 126,000 deaths per year globally [2,3]. Unfortunately, LC’s 5-year survival rate has declined from 66% to 63% over the past 40 years, despite the overall incidence of the disease decreasing [4]. Diagnosis usually occurs at an advanced stage, with roughly 60% of patients presenting with advanced disease [5]. Treatment for LC depends on tumor location, stage, patient comorbidities, and preferences, ranging from surgery alone to induction chemotherapy (IC) in selected cases. Organ preservation is prioritized in treating early-stage LC, with larynx preservation surgery (LPS) or primary radiotherapy (RT) yielding comparable oncological outcomes. Previously, total laryngectomy (TL) with postoperative RT was the standard treatment for advanced LC; however, the RTOG 91-11 study’s results shifted the treatment paradigm towards conservative laryngeal therapy with chemoradiation (CRT) [6,7]. This approach has influenced the management of advanced LC, reducing the number of primary laryngectomies. However, surgical resection remains indicated when there is radiological evidence of cartilage tumor infiltration. A combined modality approach is typically used for resectable, advanced-stage glottic and supraglottic LC [8]. If laryngeal preservation is desired, CRT is recommended, based on results from the RTOG 91-11 trial [6]. A variety of factors act in conjunction in predicting survival after diagnosis of head and neck cancers, specifically LC. Survival mainly depends on tumor stage, patient age, tumor location, cervical lymph node invasion, and a variety of other histopathological prognostic parameters [9,10,11,12]. According to the literature, it has been reported that a significant proportion of patients with primary LC develop locoregional recurrences within 2 to 3 years after completion of treatment. Specifically, studies have shown that the incidence of locoregional recurrence in this patient population ranges from 22% to 31% [7,13]. In this context, the present study aims to further contribute to the understanding of LC by analyzing the clinicopathological characteristics, treatment patterns, and outcomes of a single cohort of patients. When conducting a survival analysis of LC, it is crucial to take into account the highly heterogeneous nature of this type of cancer. Glottic tumors are typically detected at an earlier stage than supraglottic tumors, which can impact treatment options and prognosis. Therefore, it is important to perform survival analysis based on tumor stage and subsite and other potentially prognostic parameters to account for these differences [14,15,16]. If risk factors for recurrent disease can be identified, treatment can be more individualized and improved. Furthermore, follow-up visits can be more individualized based on these risk factors.

In this context, the present study aims to further contribute to the understanding of LC by analyzing the clinicopathological characteristics, treatment patterns, and outcomes of a single cohort of patients. Importantly, the study emphasizes the usefulness and importance of its findings by highlighting their potential impact on clinical decision-making and patient outcomes.

## 2. Materials and Methods

**Figure 1:**
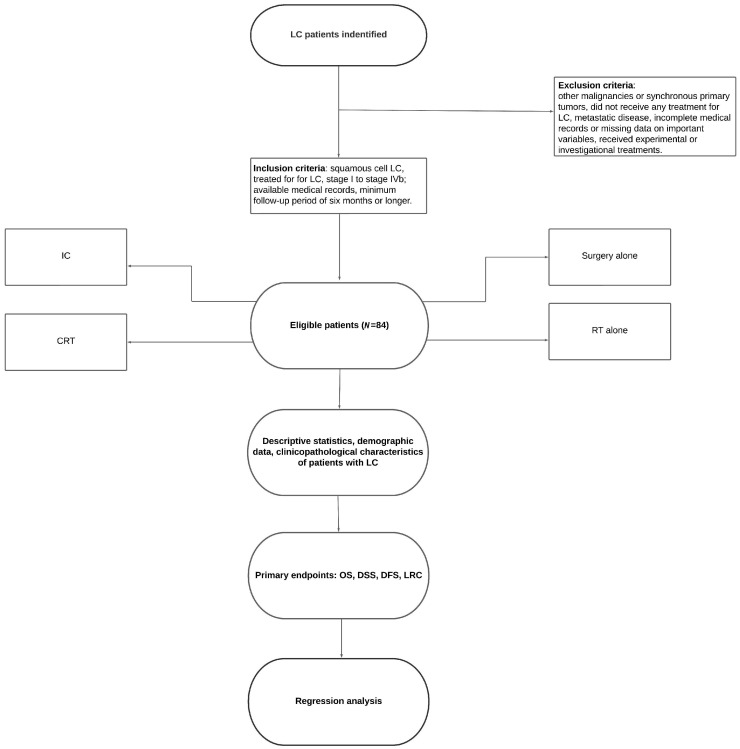
Flowchart of patient selection.

We conducted a retrospective observational study of patients with squamous cell LC treated with curative intent at the Clinic of Oncology, University Clinical Center of Republic of Srpska, Bosnia and Herzegovina (B&H), between January 2015 and December 2022. The inclusion criteria for this study were as follows: patients diagnosed with squamous cell LC confirmed by histological examination; patients who underwent treatment for LC, including surgery, RT, or chemotherapy, or a combination of these modalities; disease stage from stage I to stage IVb; available medical records, including clinical and pathological data, treatment details, and follow-up information; patients with complete data on the variables of interest, such as age, sex, smoking history, tumor stage, treatment modality, and survival outcomes; and a minimum follow-up period of six months or longer. The exclusion criteria were: a history of other malignancies or synchronous primary tumors other than basal cell carcinoma or in situ carcinomas; patients who did not receive any treatment for LC; patients with metastatic disease; incomplete medical records or missing data on important variables; and patients who received experimental or investigational treatments were excluded from the study (Figure 1). For the current analysis, the follow-up period began at the end of treatment, and the patients underwent their first clinical and radiological evaluation 4–6 weeks after completing the primary treatment. During the first year of follow-up, patients were scheduled for visits every 8–12 weeks, and subsequently, they were seen 2–3 times during the second and third years.

The disease stage was determined by using the 8th version of the American Joint Committee on Cancer (AJCC) tumor, node, and metastases (TNM) classification system [17]. Data, including patient demographic data, clinical characteristics, histopathology prognostic parameters, treatment patterns, and outcomes, were collected from medical files and entered into a database. The efficacy of therapy was evaluated according to the response evaluation criteria in solid tumors (RECIST, version 1.1) by using a computed tomography scan (CT) (Siemens Healthcare GmbH, Erlangen Germany), positron emission tomography using 18F-fluorodeoxyglucose (PET-CT) (Siemens Healthcare GmbH, Erlangen Germany), ultrasonography (US) (Sony Belgium, Zaventem Belgium), clinical examination, and laboratory tests [18]. Patients were treated with surgery, RT, CRT, or IC according to the valid recommendations for each protocol.

SPSS software version 23 (IBM SPSS Statistics for Windows, version 23.0, IBM Corp., Armonk, NY, USA).was utilized for statistical analysis of the data. Descriptive statistics were used to evaluate absolute values and percentages, while the primary endpoints were OS, DSS, DFS, and LRC. The survival rates were calculated using the Kaplan–Meier method, and the log-rank test was used to compare the results. Statistical significance was defined as a *p*-value of ≤0.05. OS was calculated from the treatment initiation date to the date of death from any cause, with patients who did not die being censored on the last available visit date in the database. In the analysis, DSS was calculated until the date of death from LC, or was censored at the date of death from non-cancerous causes or at the last follow-up. Meanwhile, DFS was calculated from the beginning of treatment until the date of recurrence or death from the underlying cancer. LRC was defined as the time interval from the start of treatment until locoregional relapse or censored at the date of death if the patient died from non-cancerous reasons without experiencing relapse. For patients who were still alive and had not experienced relapse, the LRC endpoint was defined as the last date of follow-up.

Regression analysis with the endpoints OS, DSS, DFS, and LRC was performed using the proportional hazards model. Cox proportional hazards model (forward conditional) was applied to all variables showing an effect in univariate analysis for each endpoint to determine if the variables had an independent effect on survival. Age at first diagnosis, tumor location, TNM classification, recurrence, certain histopathology parameters, smoking, and alcohol use were included as explanatory variables. The model was fed tumor location data divided into two groups: glottic and supraglottic, with subglottic carcinomas grouped with glottic carcinomas. Additionally, T and N statuses were represented as binary variables, with advanced T stage encompassing T3 and T4, and advanced N stage including patients with N1 to N3 status. Lastly, the most recently available visit date in the database was 31 December 2022.

## 3. Results

### 3.1. Sample Characterisrtics

Our study included eighty-four consecutive patients, seventy-eight men and six women, with a median age of 66 years. All of them had proven squamous cell carcinoma of the larynx. Clinicopathological characteristics are presented in more detail in Table 1. All patients were treated with curative intent. In six patients (7.2%), surgery was not performed as an initial treatment method, instead they underwent RT, CRT, or IC. Most of the patients had surgery alone or surgery combined with another treatment modality in an adjuvant setting. In 78 operable patients, we performed 49 LPS (62.8%) and 29 (37.2%) TL. After a median follow-up of 76 months, 28 (33.3%) patients had a recurrence. Local recurrences were confirmed through direct laryngoscopy and biopsy. In cases where there was doubt, all instances were restaged using US, CT, and PET-CT. Most recurrences, a total of 17 (47.2%), occurred in the largest group, surgery + CRT. Four patients (15.4%) had recurrence in the surgery group, three (25%) in the surgery + RT group, both patients in the group with IC had a recurrence, and one patient for both the RT and CRT group. The pattern of recurrence is depicted in Table 2.

### 3.2. Survival

**Figure 2:**
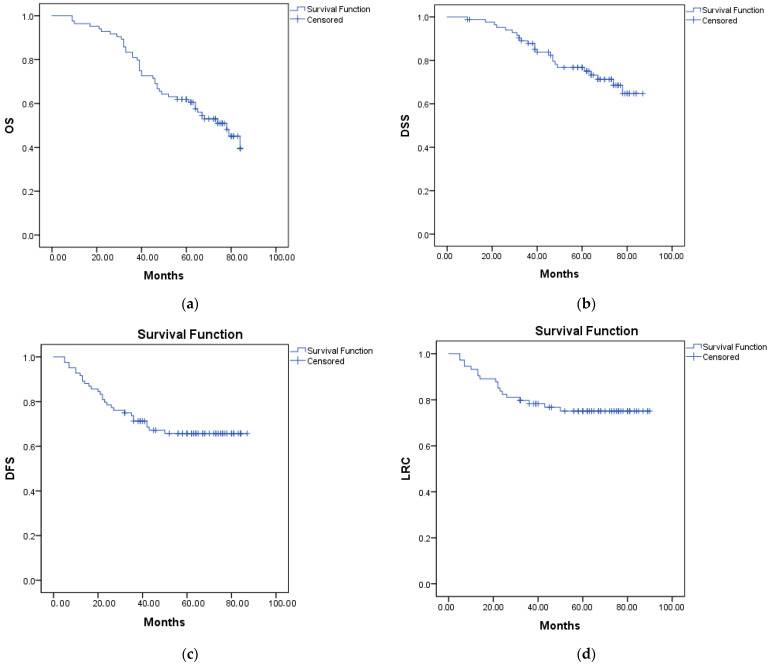
(**a**) Kaplan–Meier curve showing OS, median 78 months; (**b**) DSS, median NR; (**c**) DFS, median NR; (**d**) LRC, median NR.

**Figure 3:**
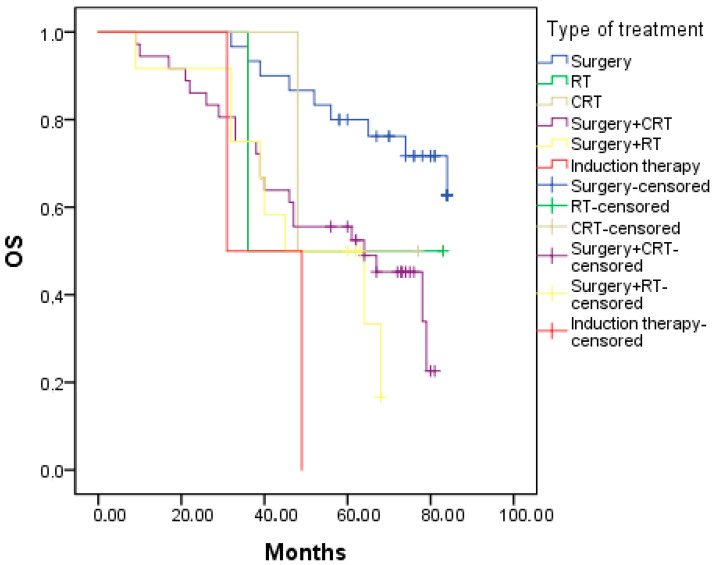
Kaplan–Meier curve showing OS in different treatment groups, log-rank *p* = 0.009.

The calculated median of OS in all patients was 78 months, 95% confidence interval (95% CI): 64.28–91.71%, with an event recorded in 50% of patients (Figure 1). DSS median was NR, with a survival rate of 72.6%. DFS survival rate was 66.7% with a median NR, and the LRC survival rate was 72.6% with a median NR (Figure 2). Table 3 shows the survival rate for specific categories. The distribution of OS, DSS, DFS, and LRC for different treatment approaches is statistically significant in most of the groups and is shown in Table 3. The Kaplan–Meier curve for OS is shown in Figure 3. The causes of death were also analyzed, and the results showed that 23 patients (27.4%) died from disease progression, 19 (22.6%) from causes unrelated to the primary disease, and 42 (50%) of the patients were alive at the end of the follow-up period.

### 3.3. Regression Analysis

Univariate analysis demonstrated that subsite location, recurrences, vascular, lymphatic, and perineural invasion, advanced T stage, advanced N stage, and gender significantly affected different types of survival (Table 4). However, multivariate analysis (Table 4) of significant variables showed that recurrence and lymph invasion were the only factors that had an independent effect on OS and recurrence in DSS. Furthermore, subsite location was the only factor in multivariate analysis that impacted DFS and LRC.

## 4. Discussion

In our hospital, the multidisciplinary team (MDT) for head and neck cancers is responsible for selecting the optimal treatment for LC. Treatment decisions for all patients with head and neck cancer, including LC, are made by this team. The larynx plays a crucial role in speech and communication; therefore, organ preservation strategies in treating laryngeal cancer are vital. It is important to note that the type of LPS used depends on the location and extent of the cancer, as well as the patient’s overall health and individual needs. A considerable number of LPS were performed in our clinical center (58.3%), but six patients were inoperable due to high disease stage, old age, and/or comorbidities. LPS included: cordectomy, endoscopic laser surgery, supraglottic laryngectomy, and hemilaryngectomy. As shown in Table 1, two patients underwent RT as a non-surgical treatment option for larynx preservation. A recent and interesting study from Romania revealed that TL was the most common surgical treatment option, accounting for 63.15% of cases [19].

In our study, the median age of the patients was 66 years, and the male predominance in the epidemiology of the disease was consistent with the findings reported in the literature [20]. This could be attributed to addiction habits that are more frequent among males [21]. Almost 90% of the patients were passionate smokers (≥2 packs of cigarettes), with 60% consuming alcohol regularly. Most of our cases were glottic carcinomas (50%). An interesting finding was that patients with vascular, lymphatic, or perineural invasion had lower survival rates and a highly significant effect on all survival categories in univariate analysis. In the multivariate models, only lymphatic invasion had an independent effect on OS. Other studies have shown that lymphatic and vascular invasion have significant prognostic value and significantly influence DFS, regional, and distant metastasis rates [22,23]. Clinicians should also consider perineural invasion because its presence is an indicator of tumor recurrence and lower survival. The presence of perineural invasion is associated with an increased risk of local recurrence and regional nodal spread, negatively affecting the prognosis of patients with LC [24,25].

Unfortunately, there is no available data regarding LC in B&H. However, we did come across a study conducted in an Ear, Nose and Throat Clinic in our country that focused in general on head and neck tumors. An analysis of 230 cases of LC between 2003 and 2007 revealed a diverging trend between the sexes. Specifically, there was a significant decrease in rates among males, while rates increased among females [26]. Since the early 1980s, LC mortality rates in men have been leveling off in most western and southern European countries, while in central and eastern Europe, this has occurred since the early 1990s. In 2010–2011, the highest male mortality rates were observed in Hungary, the Republic of Moldova, and Romania, with rates over 6 per 100,000, while the lowest rates were seen in Finland, Norway, Sweden, and Switzerland, with rates below 1 per 100,000. For women in the European Union, mortality rates remained stable at around 0.29 per 100,000 between 1980 and 1994, and slightly decreased thereafter at an average annual percent change of −1.3%, resulting in a rate of 0.23 per 100,000 in 2000–2001 [27].

After a median follow-up of 76 months, the survival rates for OS, DSS, DFS, and LRC in our study were 50%, 72%, 66%, and 72%, respectively. Studies often report a five-year median follow-up, while our research had a 76-month follow-up. Real-world studies with a five-year follow-up and a higher number of patients showed figures of 83%, 96%, 76.7%, 164, and 82–87%, respectively [16,28,29,30,31]. The Kaplan–Meier curve showed statistically significant results for treatment modality in OS, DSS, and DFS. Due to sample number disparity, regression models were not applied to the therapy type category. In advanced cases (stage III-IV), survival rates were lower, as expected, and similar to results from other studies [7,16,31]. High DSS rates indicate that the longer we follow the patients, the greater the chance of an event and death from diseases unrelated to the primary process, which is reflected in a low OS rate in our patient group. Therefore, comorbidities could play an important role in predicting OS in this patient group. Additionally, other literature data show us that comorbidities may affect diagnosis, treatment, and prognosis, and are known to be strong independent predictors of mortality in patients with head and neck cancers [32]. The prevalence of distant LC metastasis in our study was particularly high at 25%, with a predominance of lung metastasis (17.9%) and local or regional recurrence mostly present. A possible explanation is that many patients did not have a baseline chest CT. The prevalence of new primary tumors in LC was 4.8%. As previously mentioned, a large proportion of patients died from comorbidities. Adding to that, death due to a secondary malignancy must be considered when we talk about OS in patients with LC.

The Carolina Head and Neck Cancer Study (CHANCE) conducted a population-based case-control study to analyze primary HNSCC diagnosed in patients who resided in central North Carolina. The study found an overall 5-year survival rate of 59% for LC. A subsequent long-term analysis of cases that survived beyond five years after treatment suggested that initial tumor and nodal burden may be less important determinants of OS [33]. Previous studies have shown that mortality directly attributable to the initial HNSCC stabilizes after five years, but remains a leading cause of death [34]. Interestingly, the minimal impact of overall staging on OS beyond five years suggests that factors not directly attributable to the primary HNSCC, such as second primaries and cardiovascular disease, may become more important determinants of mortality [33]. In our study, we also observed an OS of 50% after seven years of follow-up. Given the good DSS rate in our study, we can concur with these findings that secondary factors significantly contribute to mortality in the long-term.

A recent study on LC conducted in our neighboring country, Croatia, reported similar results to our own findings. Specifically, the study found comparable rates of local relapse occurrence and metastases, but higher percentages of new primary cancer occurrences. However, the study did not perform survival analysis or analysis of prognostic factors [35].

Our analysis shows that survival after LC diagnosis depends on multiple factors that act in combination. Comparing survival rates across studies is challenging, as most focus on specific patient populations rather than a general sample. The development of local or regional recurrence is the most prominent risk factor for mortality in our study, along with lymphatic invasion as a pathohistological prognostic factor. As in several other studies [36,37], tumor subsite location significantly increases the hazard of mortality in multivariate analysis for DFS and LRC, and patients with supraglottic tumors have a higher risk compared to those with glottic tumors, likely due to delayed diagnosis. Advanced T stage and disease stage were significant in univariate analysis but not as independent prognostic factors. Smoking and alcohol consumption are known risk factors for both disease development and survival after LC diagnosis [38,39]. However, including these lifestyle factors did not notably change the results described above.

In a scientific paper from Pakistan, a larger cohort of patients was examined. The results of the multivariate analysis showed that nodal involvement had an independent effect on patient survival. However, the study faced several challenges, including a lack of surgical expertise in endoscopic laryngeal surgeries for early-stage diseases. As a result, many patients underwent non-surgical treatments initially, and salvage TL was offered in cases of treatment failure. Unfortunately, the refusal rate for salvage surgery was found to be high at 34%. This led to many operable diseases progressing to inoperable stages. For those patients who did undergo salvage TL, the disease recurred in around 43.3% of cases [40].

Our research is unique in B&H, as well as in neighboring countries, in that it presents the results of a thorough multivariate analysis and survival rates following a diagnosis of LC. Future research could benefit from investigating the impact of treatment on quality of life through multivariate analysis. Additionally, we may explore the effects of modifiable risk factors such as smoking and alcohol consumption reduction or cessation following a diagnosis, on survival rates in LC. In addition, this study has the accuracy of data from diagnosis until the last follow-up due to the single-center nature of the study and long observational period. However, some potential shortcomings need to be considered, such as the retrospective nature of the database, which prevents control of all variables and exclusion of biases and confounders. Additionally, the small number of patients and the possibility of under-reporting of patients managed outside the institution during follow-up should also be acknowledged. However, given the standardized clinical regimen and the cooperation with referring otorhinolaryngological departments, we do not have indications of such biases. Furthermore, advancements in diagnostic and post-treatment imaging surveillance could affect tumor classification and the ability to define recurrent cases accurately. Despite these limitations, we consider the multivariate Cox regression analysis to be the best method to present the adjusted HR of multiple risk factors in everyday clinical practice.

Considering that 56% of patients are diagnosed with a locally advanced stage of the disease, it is imperative to focus on prevention mechanisms for LC. The primary measures should be aimed at reducing smoking, alcohol consumption, and improving oral health. Additionally, regular follow-up appointments are necessary to ensure timely detection of disease relapse (surgical assessment, neck US, or CT of the neck and chest). As previously mentioned, patients were scheduled for visits every 8–12 weeks during the first year of follow-up, and subsequently, they were seen 2–3 times during the second and third years.

The TNM staging was sometimes inadequate; some patients did not receive an initial chest CT, as our study showed high percentage of lung relapses. To address this issue, we propose making chest CT a standard during the patient’s first meeting with the surgeon or during the MDT assessment. Our center offers a wide range of treatment options for LC, including LPS and TL surgical treatments, as well as intensity modulated RT and accompanying cytostatic therapy. However, we lack access to newer treatment methods for metastatic disease or locally advanced inoperable disease, such as immunotherapy. Furthermore, the use of targeted therapy may be limited due to certain constraints.

In conclusion, the unique anatomy of the larynx necessitates a specialized approach, such as organ preservation techniques. Survival trends were consistent with other studies, except for OS. Recurrence, lymphatic invasion, and subsite location were significant factors that impacted patient survival. Further research is required to understand the biological, genetic, and environmental interactions at play.

## Figures and Tables

**Table 1:** Clinicopathological characteristics and survival rates for all patients (*N* = 84).

Characteristic	Category	*N* (%)	OS (%)	DSS (%)	DFS (%)	LRC (%)
Gender	Men	78 (92.9)	50	70.5	64.1	70.5
	Women	6 (7.1)	50	100	100	100
Age	50–<60 years	19	42.1	63.2	68.4	84.2
	60–<70 years	42	61.9	76.2	64.3	66.7
	70+ years	23	34.8	73.9	69.6	73.9
Subsite	Glottic	42 (50.0)	66.7	90.5	83.3	83.3
	Supraglottic	39 (46.4)	30.8	53.8	48.7	61.5
Surgery	LPS	49 (58.3)	57.1	85.7	77.6	77.6
	TL	29 (34.5)	41.4	58.6	55.2	69
	Inoperable	6 (7.1)	33.3	33.3	33.3	50.0
Surgical margin	Negative	56 (66.7)	55.4	80.4	73.2	75
	Positive	21 (25.0)	38.1	61.9	57.1	71.4
	Not evaluable	7 (8.3)				
Tumor grade	Grade 1	15 (17.9)	73.3	73.3	66.7	73.3
	Grade 2	58 (69.0)	81.0	74.1	67.2	74.1
	Grade 3	11 (13.1)	72.7	63.6	63.6	63.6
Vascular invasion	Not present	44 (52.4)	61.4	77.3	75	79.5
	Present	33 (39.3)	36.4	60.6	51.5	60.6
	Unknown	7 (8.3)				
Lymph invasion	Not present	38 (45.2)	61.4	77.3	72.7	77.3
	Present	39 (46.4)	36.4	60.6	54.5	63.6
	Unknown	7 (8.3)				
Perineural invasion	Not present	42 (50.0)	63.2	56.4	78.9	78.9
	Present	35 (41.5)	38.5	45.2	51.3	64.1
	Unknown	7 (8.5)				
Smoking	Yes	74 (88.1)	52.7	71.6	74.3	78.5
	No	10 (11.9)	30.0	80.0	60.0	55.6
Alcohol	Yes	51 (60.7)	52.9	76.5	74.5	78.4
	No	33 (39.3)	45.5	66.7	54.5	63.6
T stage	T1	14 (16.7)	78.6	92.9	78.6	78.6
	T2	36 (42.9)	47.2	72.2	75.0	77.8
	T3	16 (19)	37.5	75.0	62.5	62.5
	T4	18 (21.4)	44.4	55.6	44.4	66.7
N stage	N0	56 (66.7)	55.4	78.6	73.2	76.8
	N1-3	28 (33.3)	39.3	60.7	53.6	64.3
Disease stage	I	14 (16.7)	78.6	100.0	78.6	78.6
	II	23 (27.4)	47.8	73.9	82.6	82.6
	III	22 (26.2)	50.0	86.4	77.3	77.3
	IVa, IVb	25 (29.8)	36.0	44.0	36.0	56.0
Therapy	Surgery + CRT	36 (42.9)	41.7	61.1	52.8	63.9
	Surgery	30 (35.7)	70.0	96.7	86.7	86.7
	Surgery + RT	12 (14.3)	33.3	66.7	75.0	75.0
	RT	2 (2.4)	50.0	50.0	50.0	50.0
	CRT	2 (2.4)	50.0	50.0	50.0	50.0
	IC	2 (2.4)	0.0	0.0	0.0	50.0

**Table 2:** Disease recurrences and second primary malignancies in all patients, *N* = 84.

Site of Recurrence	*N*	%
Local recurrence	18	21.4
Lung	15	17.9
Lymph nodes	13	15.5
Bones	4	4.8
Liver	2	2.4
**Incidence of second primary malignancy**	4	4.8
Lung cancer	2	2.4
Rectal cancer	1	1.2
Acute myeloid leukemia	1	1.2

**Table 3:** Survival for different treatment groups.

Statistics	Surgery	RT	CRT	Surgery + CRT	Surgery + RT	IC	Overall
**OS (months)**	NR	36	48	64	45	31	78
*N*	30	2	2	36	12	2	84
*N* of events	9	1	1	21	8	2	42
95% CI				40.1:87.9	17.8–72.1		64.3–91.7
*p* value							0.009
**DSS**	NR	36	48	78	NR	31	NR
*N*	20	2	2	36	12	2	84
*N* of events	1	1	1	14	4	2	23
95% CI				56.3:94.8			
*p* value							0.001
**DFS**	NR	35	42	NR	NR	13	NR
*N*	30	2	2	36	12	2	84
*N* of events	4	1	1	17	3	2	28
95% CI							
*p* value							0.022
**LRC**	NR	35	42	NR	NR	13	
*N*	30	2	2	36	12	2	84
*N* of events	4	1	1	13	3	1	23
95% CI							
*p* value							0.297

**Table 4:** Results from univariate and multivariate analyses.

	**OS**						**DSS**					
**Characteristics**	**HR (a)**	**95% CI**	***p* Value**	**HR (b)**	**95% CI**	***p* Value**	**HR (a)**	**95% CI**	***p* Value**	**HR (b)**	**95% CI**	***p* Value**
Surgery							3.2	1.3:8.2	0.014			
Subsite	3.1	1.6:5.9	0.001				7.1	2.4:21.2	<0.001			
Recurrences	5.0	2.7:9.3	<0.001	5.6	2.8:11.2	<0.001	35.5	8.3:152	<0.001	22.5	5.1:99.6	<0.001
Vascular invasion	2.1	1.1:3.9	0.026									
Lymph invasion	2.2	1.1:4.2	0.015	2.1	1.1:4	0.031	2.3	1:5.3	0.048			
Perineural invasion	1.2	1.02:3.8	0.043				3.2	1.3:8.2	0.013			
Advanced disease stage							2.7	1.1:7.0	0.033			
Advanced N stage							2.3	1:5.2	0.047			
	**DFS**						**LRC**					
**Characteristics**	**HR (a)**	**95% CI**	***p* Value**	**HR (b)**	**95% CI**	***p* Value**	**HR (a)**	**95% CI**	***p* Value**	**HR (b)**	**95% CI**	***p* Value**
Gender	2.2	1.0:5.0	0.045									
Subsite	4.2	1.7:9.9	0.001	4.5	1.8:11.3	0.001	2.9	1.2:7.2	0.018	3.1	1.2:8.2	0.017
Vascular invasion	2.3	1.1:5.1	0.028				2.3	1.1:5.0	0.031			
Perineural invasion	2.7	1.2:6.2	0.018				2.6	1.1:6.0	0.021			
Advanced T stage	2.1	1.0:4.6	0.042									
Advanced stage	2.8	1.2:6.7	0.016				2.7	1.1:6.5	0.020			

a: Results from univariate analysis; hazard ratio (HR); 95% CI; *p* value. b: Results from multivariate analysis; HR; 95% CI; *p* value.

## Data Availability

The data presented in this study are available on request from the corresponding author. The data are not publicly available due to privacy and ethical issues.
